# The Contraceptive Pill in Ireland *c.*1964–79: Activism, Women and Patient–Doctor Relationships

**DOI:** 10.1017/mdh.2020.3

**Published:** 2020-04

**Authors:** Laura Kelly

**Affiliations:** School of Humanities, University of Strathclyde, 141 St James Road, Glasgow G4 0LT, UK

**Keywords:** Contraception, Birth control, Ireland, Contraceptive pill, Catholicism

## Abstract

The twentieth-century history of men and women’s attempts to gain access to reproductive health services in the Republic of Ireland has been significantly shaped by Ireland’s social and religious context. Although contraception was illegal in Ireland from 1935 to 1979, declining family sizes in this period suggest that many Irish men and women were practising fertility control measures. From the mid-1960s, the contraceptive pill was marketed in Ireland as a ‘cycle regulator’. In order to obtain a prescription for the pill, Irish women would therefore complain to their doctors that they had heavy periods or irregular cycles. However, doing so could mean going against one’s faith, and also depended on finding a sympathetic doctor. The contraceptive pill was heavily prescribed in Ireland during the 1960s and 1970s as it was the only contraceptive available legally, albeit prescribed through ‘coded language’. The pill was critiqued by men and women on both sides of the debate over the legalisation of contraception. Anti-contraception activists argued that the contraceptive pill was an abortifacient, while both anti-contraception activists and feminist campaigners alike drew attention to its perceived health risks. As well as outlining these discussions, the paper also illustrates the importance of medical authority in the era prior to legalisation, and the significance of doctors’ voices in relation to debates around the contraceptive pill. However, in spite of medical authority, it is clear that Irish women exercised significant agency in gaining access to the pill.

## 1 Introduction

In a book entitled *Marriage Irish Style*, published in 1969, journalist Dorine Rohan interviewed married Irish men and women about their lives. Her book provided an insight into Irish marriage and family life and illuminated a huge degree of sexual ignorance, the perceived sinfulness of sex, as well as domestic abuse.^1^ In a chapter on religion in Irish marriages, she wrote of one woman whose account was typical of many of the women she met.^2^ The woman was pregnant for the third time in three years and her doctor had admitted her to hospital because he was concerned that she would lose the baby because of her poor living conditions:

We live with my mother. My husband, myself and the two kids. We had a flat but it was too expensive; now we only have one room for the four of us. I’d like to have spaced the children better. When I told the priest I was expecting this one he said I should have been more careful. My husband leaves for work in a temper every morning from the set-up. We have put our name down for a council house, but they said we’d have a better chance if we had more kids. I don’t think I could have any more for a while after this one. I’ll try for the pill, if not we’ll just have to try something else, won’t we? I would rather keep up my religion, but I’ll have to do something this time.^3^

The woman’s testimony highlights the difficulties she faced under the contraceptive ban; the impact this had on her marital relationship, the role of the Church in her reproductive choices and her moral dilemma about going on the contraceptive pill. While the woman interviewed does not explicitly judge the priest for his comments, they illustrate his belief that family planning was her responsibility, while her remark about ‘trying for the pill’ shows that it was not guaranteed that she would find a doctor to prescribe it for her. For this woman, taking the contraceptive pill was necessary in order to better improve her family situation. Such testimony was not unusual and frequently utilised by advocates for the legalisation of contraception in Ireland. Women’s magazines in the 1960s and 1970s highlighted such cases. An article in *Woman’s Way* magazine in 1969, for instance, interviewed Marie Monaghan, aged twenty-four, and the mother of six children, the youngest children being four-month-old triplets. Monaghan explained:

I certainly don’t want any more children; I’ve had enough. My doctor has promised to put me on the Pill and I won’t have any qualms at all about using it. People can sermonise as much as they want to about what the Pope said in the encyclical and so on, but how do you look after your large family when your husband is unemployed and the bills are mounting up?^4^

Monaghan’s account here justifies her going against Catholic doctrine and taking the contraceptive pill for economic reasons, and like Rohan’s respondent, the pill was viewed as necessary in order to better her family situation. Such testimonies suggest that some Irish women were finding ways to navigate both the legislative and religious restrictions on contraception, and that they did not necessarily have qualms about doing so.^5^

Contraception was made illegal in the Republic of Ireland in 1935 following the introduction of the Criminal Law Amendment Act.^6^ The ban, as Sandra McAvoy has pointed out, arguably ‘delayed the emancipation of Irish women – not least by subordinating their rights to life and health to their reproductive functions’.^7^ Contraception was not legalised until 1979 when the Family Planning Act was introduced which allowed contraception on prescription for *bona fide* family planning purposes only, with this stipulation widely interpreted as meaning that contraceptives were only available to married couples. This legislation was referred to by the minister for health, Charles Haughey, as an ‘Irish solution to an Irish problem’. Recent valuable studies have highlighted the role of the Catholic Church hierarchy, the Irish government and the medical profession in debates surrounding contraception in Ireland in the twentieth century.^8^ Lindsey Earner-Byrne, for instance, has shown how in the twentieth century ‘many in the medical profession were deeply influenced by Catholic social teaching and used papal teaching to bolster their arguments against any form of state medicine and to promote Catholic control of medicine’.^9^ Similarly, in Northern Ireland, artificial birth control was unavailable and largely condemned until the 1960s and there was significant opposition from the Catholic Church and politicians there to the establishment of family planning services.^10^ Important studies by Connolly, Hug, Cloatre and Enright have illuminated the campaigns of feminist and activist groups to legalise contraception.^11^ Significant work by Earner-Byrne, Rossiter, and Earner-Byrne and Urquhart have also highlighted the consequences of lack of contraceptive and abortion options in Ireland, and in particular highlighted the pattern of travel to England for reproductive healthcare.^12^ However, with regard to the history of contraception in Ireland, the experiences of ‘ordinary’ women have been neglected and there has been more focus given to the role of the medical profession and institutional reform.^13^ Two important exceptions are Betty Hilliard’s groundbreaking study of Irish married women, which draws on interviews with 105 women in Cork City in 1975, and a number of repeat interviews in 2000, and Máire Leane’s study of Irish women’s sexual knowledge and sexual experiences which utilised twenty-one interviews with women born between 1914 and 1955. These studies illuminate the relationship between Irish women and the Roman Catholic Church, in particular focusing on changing attitudes to sexuality and the use of birth control.^14^

This article therefore seeks to build on important recent accounts of the contraceptive pill in other predominantly Catholic countries with similar restrictions in place and ultimately to contribute to our understandings of the history of contraception in modern Ireland.^15^ I will focus on three key themes: firstly, the experiences of Irish women who chose to take the contraceptive pill; secondly, the role of medical and papal authority surrounding the pill; and finally, debates around the pill from feminist and anti-contraception campaigners. Ultimately I seek to show here that through negotiating access to the contraceptive pill, Irish women were also negotiating their Catholicism, marriage dynamics, and relationships with the medical profession and the priesthood. Drawing on women’s magazines, memoirs, archival sources, newspapers and oral history interviews, the article seeks to show the importance of class and economic factors in debates around the contraceptive pill, and how the contraceptive pill became an important symbol of the debate around contraception in 1960s and 1970s Ireland.^16^ For instance, contraception was discussed regularly in *Woman’s Way* magazine, with readers’ letters on the subject being published from 1966, following the lead-up to the papal encyclical and an article by journalist Monica McEnroy on the Pope and the Pill in 1966 that ‘sparked so many responses that a special page was given over to them’.^17^ Women’s accounts of the contraceptive pill became an important element of debates in the printed media around contraception in the 1960s and 1970s. This was in part because the fact that the contraceptive pill was dissociated from the act of sexual intercourse meant that it became an easier vehicle for discussion, unlike, for instance, condoms, which continued to have an association with sexually-transmitted diseases.^18^ For both feminist and anti-contraception campaigners, the side effects of the contraceptive pill became a focal point of campaigning.

## 2 ‘An Unstated Contract’: Women’s Agency and Doctors’ Authority

The contraceptive pill was available in Ireland on prescription from 1963 and marketed as a cycle regulator, and, as Mary E. Daly has argued, ‘played a crucial role in opening a debate on contraception’.^19^ Users of the contraceptive pill could circumvent the ban on contraception in Ireland by asking for the pill as a cycle regulator rather than as a contraceptive. In Spain, where contraception was banned until 1978, the contraceptive pill was marketed as an ‘oral cycle regulator’ or as an ‘ovulostatic’ and, during the 1970s, marketing materials and package inserts continued to inform patients that these drugs ‘should be used to allow for “periodic rest of the ovaries”’.^20^ The situation was similar in Ireland. For instance, in a 1967 edition of the *Journal of the Irish Medical Association*, an advertisement for oral contraceptive Lyndiol 2.5 advertised the drug ‘for a menstrual cycle as regular as clockwork’ and advised that ‘the use of a combined oestrogen/progestogen tablet is now generally accepted as the most suitable method of treatment’.^21^ The advertisement shows a woman’s hand with a watch; the woman is wearing a wedding ring. As Elizabeth Siegel Watkins has illustrated, by the mid-1960s, the use of the contraceptive pill and other contraceptive methods by single women was frowned upon ‘because it implied, correctly, not only that these women were having sex but also that they were planning ahead for it’.^22^ Similarly, in the UK, the prescription of the contraceptive pill to unmarried women was disapproved of and in the first years of its introduction to the UK, the contraceptive pill was restricted to married women. Family Planning Associations only provided contraception to married or engaged women, while the 118 government-funded local authority clinics restricted provision of contraception to married women who required it on ‘medical grounds’, while many general practitioners (GPs) believed that contraception should not be part of their medical practice.^23^ Furthermore, GPs who did provide contraception usually did not prescribe it to single women. However, some women found ways to circumvent this, through attending a sympathetic GP, or lying about their marital status.^24^ From the 1960s, this practice was occurring within a wider context of increasing patient autonomy and consumerism.^25^ From 1964, the contraceptive pill was available in Britain to unmarried women through Brook clinics.^26^ In 1968, the Family Planning Association gave permission to their branches to prescribe the pill to unmarried women also; their branches were required to do so from 1970.^27^

By 1967, Syntex Pharmaceuticals, the manufacturers of more than half of the contraceptive pill brands, estimated that 12 000 Irish women (three out of every hundred) were taking the pill, and by the following year, this was estimated to have risen to five out of every hundred.^28^ Between 1966 and 1967, it was estimated that there was a fifty per cent increase in the usage of the pill in Ireland, with four anovulent brands being available in 1966, and at least ten in 1967.^29^ Given that the contraceptive pill was the only artificial contraceptive available from general practitioners in Ireland pre-legalisation, albeit through the use of coded language, it became an important emblem in debates around the legalisation of family planning in the 1960s and 1970s. As in Britain, where general debate about access to contraception was increasingly about access to the pill. The word ‘pill’ was frequently used in the press as a synonym for contraception and vice versa by the early 1970s.^30^ For middle- and upper-class men and women based in Dublin, contraception could be obtained from 1969 through the Fertility Guidance Company, later the Irish Family Planning Association (IFPA), a clinic which circumvented the law on contraception by asking patients for a ‘donation’ for contraceptives.

Professor Dermot Hourihane (b.1933), a professor of pathology and one of the founder members of the Fertility Guidance Company, explained in an oral history interview to me:

What the pill did was, first of all, it was a first class contraceptive, and most of all, you took it orally, so you didn’t have to put anything in or out, so there was no intrusion into your body. That made it acceptable to all Irish people. There was no feeling of this horrible, dirty thing, and putting on, or whatever. It was socially acceptable…so then, of course, what happened was the church said the pill was okay for regulating irregular periods, so all the well-to-do women that could pay the doctor said, ‘I’d like the pill, please, my periods are very irregular’, and he would say, ‘Right’, with a nod and a wink sort of thing. That was an unstated contract, almost between the doctor and the…So it just made it more and more unfair, but it made contraception – it changed it from being alien into being more acceptable to an Irish woman or man.^31^

As Hourihane’s testimony suggests, oral contraceptives often appealed to women because of their reliability and independence from the act of sexual intercourse, while they were not interruptive or messy compared to other forms of artificial contraceptives.^32^ Moreover, the popularity of the contraceptive pill must be understood within the broader historical context where new pills were becoming available on prescription to treat a variety of health issues.^33^ The pill could also be bought in six-month supplies and potentially taken without the male partner’s knowledge.^34^ In Hourihane’s view, the pill evidently had an important role in the debates around family planning; however, there were important class disparities in terms of access. As John Horgan surmised in 1970, in *Fortnight* magazine, ‘the fact that the pill is much more freely available to the fee-paying middle-class patients of doctors in private practice than to the working-class mothers who have no option but to attend Church-controlled maternity hospitals, introduces an ugly element of class distinction into a situation already reeking of contradiction and hypocrisy’.^35^

Women’s personal accounts of the contraceptive pill in Ireland in the period before legalisation tend to emphasise three key themes: the first being the power of doctors over women’s reproductive choices in the period before legalisation; second, women’s beliefs that they were taking the pill in good conscience; and third, the importance of class. For many Irish women, the act of taking the contraceptive pill represented circumvention of medical, legal and religious authority. However, as many articles in the printed press drew attention to, as with other forms of contraception, there was an important class divide in terms of access.

In feminist magazine, *Wicca*, published in 1977, Ann O’Brien discussed how her decision to go on the pill after moving into a flat and deciding ‘that I wanted to enjoy myself and feel safe’. In contrast to other forms of contraception, such as condoms and the cap, she felt that the pill had an advantage of reliability and ‘because the Pill is oral it is inclined to be separated from sexuality, which in part explains its success in a sexually repressed country like Ireland, but also it means there is no temptation not to use it involved, it’s just a matter of remembering to take it all the time whether active sexually or not’.^36^

Accounts of the contraceptive pill described it in contrast with natural methods of birth control such as the rhythm method. Máire Mullarney, for instance, who was involved in the foundation of the Fertility Guidance Company, wrote in 1992:

We agreed that with this blessed Rhythm, by the time the ‘safe period’ arrived we wished sex had never been invented. You see, if you were well-informed, as we were, you knew that, not only must the husband not ejaculate, but the wife must not allow herself to experience orgasm. This while sharing the same bed. So different with the magic Pill; I could say, ‘Well, not tonight, if you don’t mind, but tomorrow will be fine.’ And it would be. Formerly there used to be the waiting and wondering, would a period ever happen? And a husband depressed for months when, after all our care, I was pregnant again.^37^

For Mullarney, the contraceptive pill provided a much needed respite from the anxiety of worrying about falling pregnant, in contrast with the rhythm method, which restricted sexual intercourse to infertile days. The rhythm method was problematic for many couples. As Dr Dermot MacDonald and Dr Declan Meagher, two doctors who ran a family planning clinic at the National Maternity Hospital in Dublin, explained in a 1967 article, the rhythm method required couples who had recently had a baby to abstain from intercourse until regular menstruation returned, which could be five to six months after birth in some cases.^38^ This, combined with the fact that couples were also advised to refrain from intercourse in the last two months of pregnancy, meant that the rhythm method imposed ‘an intolerable strain on many marriages’.^39^ Clare, a 28-year-old mother of four children, explained in an interview with *Woman’s Way* magazine in 1973 how she had tried the ‘safe period’ but found it to be ‘utterly useless’, stating: ‘Can you imagine how hard it is to confine yourself to a few days in the month when it’s safe to make love, then rigidly stick to those days? It leads to tension and frustration and makes for anything but a happy home.’^40^

Annual reports from the IFPA illustrate the prevalence of the contraceptive pill as a family planning method for IFPA patients at Dublin clinics. In 1972, for instance, 47.1% of patients at the IFPA Merrion Square clinic and 48% at the Mountjoy Square clinic were prescribed an oral contraceptive. In 1973, 57% of Merrion Square and 37% of Mountjoy Square patients were prescribed an oral contraceptive. By 1974, this had increased to 66% in the Synge Street branch (formerly Merrion Square), and 48% in Mountjoy Square, increasing to 68.25% for Synge Street in 1976 and 59.16% for Mountjoy Square in 1976.^41^ Contemporary newspaper accounts suggest that the contraceptive pill was also being readily prescribed by general practitioners in Ireland – this often involved doctors making a private agreement with patients. In 1968, the marketing director of Syntex Pharmaceuticals, Ronald Levin, stated that from ‘the conversations we’ve had with doctors in the Republic…that the majority of general practitioners in Ireland are prescribing the Pill for social reasons’.^42^ One Dublin gynaecologist explained to journalist Mary Maher in March 1968 that ‘more and more general practitioners are prescribing it, and very few doctors would refuse it now to any woman who asks for it’.^43^ Another pharmaceutical company representative stated that he believed that 25% of Irish women taking the pill were using it for ‘medical reasons’, and 75% for ‘social reasons’, with the firm’s spokesperson joking ‘Either that or there’s a great increase in menstrual difficulties’.^44^ However, the spokesperson quickly acknowledged that the company was strictly adhering to Irish legislation around contraception and that ‘chemists are just as strict about following prescriptions exactly’, stressing that ‘the great majority of doctors are deeply concerned and very anxious for a decision from Rome’.^45^ *Humanae Vitae* came at a crucial moment in terms of the history of birth control – the question of contraception had become the topic of heated debate, particularly with the advent of the contraceptive pill in Europe from the early 1960s. For many Catholics, it was hoped that the Pope’s encyclical would constitute a more relaxed approach to the issue of birth control; however, *Humanae Vitae* reinforced the Church’s views relating to the purpose of marriage and condemned all methods of artificial contraception.^46^ This meant that ‘the large number of Catholics who had started to practise birth control, sometimes with the moral support of their priest, would now have to readopt the traditional teaching or ignore the papal declaration’.^47^

As a result of the lack of other contraceptive options, some sympathetic general practitioners prescribed the pill to women who had experienced numerous pregnancies. Writing in the *Journal of the Irish Medical Association* in 1969, Dr Declan Meagher highlighted the difficult position that the Irish ban on contraception posed for doctors. He argued that the role of the doctor was to bring ‘sympathy and understanding’ to couples with problems controlling their fertility. Meagher believed that the primary responsibility of doctors was to decide on the best medical treatment for the patient. He argued that for some Irish patients and doctors, *Humanae Vitae* was not an infallible statement.^48^ Similarly, as Agata Ignaciuk’s work has shown, in Spain, *Humanae Vitae* was largely irrelevant for doctors who supported family planning and who were involved in early birth control clinics.^49^ In Meagher’s view:

It may be difficult for them to see it is immoral for man to deliberately induce a condition which nature itself produces constantly throughout the infertile days, or to turn a deaf ear to the over-burdened generous mother with five children under 7 at loggerheads with husband, children and religion who pleads ‘But doctor, is it for the good of the family?’^50^

Meagher’s testimony highlights the difficult dilemma faced by some Irish doctors, and the use of a case of a woman with multiple children as an example, was typical of evidence in favour of contraception being legalised at the time. However, it was clear that certain doctors were unsympathetic to such cases and refused to prescribe the contraceptive pill at all. Writing in *Woman’s Way* magazine in 1966, journalist Monica McEnroy drew attention to the plight of many Irish women who were unable to avail of contraception as a result of the dual legal and Catholic bans on it. Following newspaper correspondence on the pill, McEnroy had received letters from women all over Ireland ‘who had asked their doctors for the Pill and had been refused’.^51^ One woman, writing to McEnroy explained:

I am thirty-eight. I have five lovely children. The eldest is eight and I have just lost another baby before its time. I have high blood pressure for the past two years. I asked my doctor could I not try the Pill as I want to try and look after the children. The worry of another miscarriage is always hanging over me, but he told me I would have to wait until they got word from Rome.^52^

McEnroy argued that women should be allowed to decide for themselves with regard to birth control, and that the religious aspect was ‘a matter between me and God’.^53^ Similarly, in another article in *Woman’s Way* magazine in 1968, which described the experiences of women and family planning in Ireland, McEnroy interviewed a woman called Mrs Kearney, the mother of three children, who had been refused the contraceptive pill by her doctor. According to McEnroy, Kearney wanted ‘to have the same facilities for living her married life in peace and harmony with her husband and three children as her sister in England’, and she argued that ‘No hospital has the right to make me obey these regulations. I am the one to decide what is necessary for my family’.^54^ Doctors therefore had significant authority in deciding who could be prescribed the pill. Similarly in Spain, the circulation of the pill in the 1960s and early 1970s helped to ‘reinforce the doctor’s technical and gender power position’.^55^

Single women may have had particular difficulty in gaining access to the contraceptive pill from general practitioners in Ireland. Indeed, as Eimer Philbin Bowman’s 1977 study of first time visitors to a Dublin family planning clinic showed, some doctors ‘were in general unhappy about prescribing the pill for any length of time to an unmarried woman, with one respondent explaining, ‘He said he would give it to me for three months for irregularity but if I wanted it again I would have to go somewhere else’.^56^ Furthermore, there were cases where husbands interfered in women’s access to the contraceptive pill. One mother of four, writing to *Woman’s Way* advice column in July 1968 explained that her doctor had stopped prescribing her the pill because her husband had ‘called in to object…on the grounds that “you have to take what is before you in life”’. The agony aunt stated ‘I think that both your doctor and your husband have forgotten that you are the person to decide. I suggest that you make this point quite firmly and cheerfully’.^57^ Others did not tell their husbands they were taking the contraceptive pill for fear of causing an argument or tension in their marital relationship.^58^

Doctors clearly had significant power in choosing whether they would prescribe a woman the contraceptive pill; however, it is clear that women also exhibited agency in finding a sympathetic doctor who would. Knowledge of sympathetic doctors, and information on what to say, was usually spread through word-of-mouth. Similarly, Leanne McCormick’s important work on abortion in Belfast has shown the significance of women’s networks in the transmission of knowledge about illegal abortion and the restriction of such networks of knowledge within Protestant dominated neighbourhoods.^59^ For instance, as Irish feminist campaigner Ruth Riddick, who would go on to establish the Open Door Counselling service for women experiencing crisis pregnancies, explained in an oral history interview to me:

Now, needless to say, the Irish solution to an Irish problem was in place long before Charlie Haughey ever mentioned it. I remember being told by girlfriends what it is you said to which doctor to get put on the pill. That was relatively common knowledge. The pill, at this point, had been introduced since 1960. Now that we had television, now that our communication systems were working bigger and better we knew about the pill. The question only became where to get it?^60^

Similarly, the Fine Gael politician and member of the Irish Women’s Liberation Movement, Nuala Fennell, wrote in her 2009 memoirs that ‘women who identified understanding and helpful general practitioners passed on the word to other women’.^61^ However, Joanne O’Brien, a member of feminist activist group Irishwomen United (IWU), explained in an oral history interview to me that while ‘There was information shared between women about who you could go to or whatever…I think you had to have a fair amount of self-confidence to go in and ask for something like that’.^62^ Given the ban on contraception in Ireland, once a potentially sympathetic general practitioner was identified, women who wished to obtain the contraceptive pill from their general practitioner usually used the excuse of having menstrual irregularities. According to oral testimony from Betty Purcell, a member of IWU:

The cycle regulator was the thing [justification/excuse] that was used, that you would say, ‘My periods are, you know, all over the place and, you know, I just need to regularise them because they come very heavily’ or whatever. The doctor would say, ‘Oh yeah. Cycle regulator. The pill.’ That was the commonest way in which it was given out, definitely, by ordinary doctors. Yeah.^63^

Ongoing oral history research will also help to illuminate women’s personal experiences and how women obtained access to the contraceptive pill. Maria, born in 1951, from a working-class background in Co. Limerick, went to university in Galway to study for an arts degree. Her account suggests the prevalence of the withdrawal method among her peer group; however, she was aware, through her friends and boyfriend, of a sympathetic doctor she could go to in order to obtain the contraceptive pill. She explained to me in an oral history interview:

*And then in college, what kind of methods do you think people would’ve been using [for contraception]?*

Withdrawal.

*Yeah?*

Largely…I would say. Largely. Condoms were very hard to get, really. And I suppose even with myself, I mean, I was due to go on the pill, I obviously got pregnant – Seán was born in July – so I got pregnant some time around September. And I was due to go on the pill, John and I were affectively in a relationship, and I was due to go on the pill, and I was waiting for my period to come, and then I ended up going to the doctor because I was pregnant, rather than, you know. So it was either, you could get the pill, as a single woman then. You would, you would’ve been able to get the pill with some doctors, obviously some wouldn’t have given it to you.

We had made an appointment to go and see John’s doctor in Galway, I don’t think I had a doctor in Galway, he had ’cause he was there a few years before me. And then I ended up going to him because I was pregnant.

*And so did you have an idea that he would be a doctor who would prescribe the pill?*

Yes. Well, John had, that he would, yeah. As I said, not all of them would have at the time.^64^

Betty Hilliard’s study of 105 women in Cork city suggested that the women in her sample relied on ‘combinations of luck, natural methods and their partner’s co-operation to curtail their number of pregnancies’.^65^ However there is evidence to suggest that some women from working-class backgrounds possessed knowledge of how they might gain access to the contraceptive pill. Marian Larragy, a member of the Contraception Action Programme (CAP), an offshoot of feminist group IWU which illegally dispersed contraceptives and information on contraception from 1976, recalled visiting the residents of flats in Ballymun, one of Dublin’s poorer areas, in order to gain signatures for a CAP petition. She recalled meeting a young mother who had been in her class in primary school who ‘signed the petition and told me that everybody in the flats was getting the pill “to make their periods regular”’.^66^

Class was an important element of debates around access to family planning, and articles in favour of the legalisation of contraception tended to focus on ‘hard cases’ of women burdened by multiple pregnancies. For instance, ‘Layman’, the author of a letter to the *Irish Times* in 1966, described himself as a ‘member of a voluntary lay charitable organisation working mainly in the poorer areas of the city’ and outlined four problems he had encountered in the last six months, including: a ‘a woman expecting her eighth child. Her eldest child is  years. I advised her to attend a marriage guidance clinic at a city hospital. They asked her to prepare a temperature chart and return in four months. She was pregnant by then’; a woman who greeted him with the news ‘that this was her second Christmas free of pregnancy in her 17 years of marriage’; a mother of three children whose father refused to work – ‘as she does not wish to have any more children she refuses to sleep with her husband. Her confessor states she is committing mortal sin’; and a mother of twelve children who had been admitted to a mental hospital for up to four months prior to and up to four months after each birth.^67^

Middle-class men and women could navigate the legislation if they had access to a family planning clinic or had the knowledge of a sympathetic doctor or the language to use in order to obtain a prescription to the contraceptive pill. Deirdre Foley’s recent important study of family planning in Dublin in the wake of *Humanae Vitae* suggests that some Catholics who attended private family planning clinics chose to ‘quietly ignore *Humanae Vitae* and use artificial means of birth control’.^68^ In addition, it was possible for some men and women to travel over the border to Northern Ireland to obtain contraception and illegally bring it back to the Republic; the Irish Women’s Liberation Movement (later IWLM) Contraceptive Train protest highlighted this hypocrisy.^69^ However, for the majority, access to contraception was difficult. According to Monica McEnroy, writing in *Woman’s Way* magazine in 1968: ‘Unfortunately there are many women who want to avail of effective contraception control. Because they are rather poor or because they live in a country area, they have no hope of availing of the undoubted benefit of freedom from worry about pregnancy.’^70^ For Ruth Riddick, like many other women involved in campaigns around the legalisation of contraception, this injustice with regard to access to contraception prompted her to become involved in campaigning. She explained to me:

It wasn’t so much a question of could I get access to contraception or not? I could. It was more about the principle of the thing. The fact that you can sneak around and use coded language isn’t good enough in that contraception needed to be legalised.^71^

For Catholic women, the decision to take the pill involved reconciling one’s personal choices with one’s religious beliefs, and the teachings of the Church hierarchy.^72^ For the 105 women interviewed by Betty Hilliard in 1975, ‘a central issue in these women’s lives was their belief that avoidance of pregnancy was viewed by the Catholic Church as sinful: sex was for procreation’.^73^ On 28 March 1971, in response to increasing public discussion of the issue of contraception in Ireland and the activities of legislators such as Mary Robinson who were attempting to have the law liberalised, the archbishop of Dublin, John Charles McQuaid, a senior figure in the Irish Church hierarchy, had a pastoral read at every Sunday mass in the Dublin diocese. The pastoral, which was also published in Irish newspapers, declared that if legislation was passed allowing contraception, it would be ‘an insult to our Faith; it would, without question, prove to be gravely damaging to morality, private and public; it would be and would remain a curse upon our country’.^74^ Furthermore, he highlighted the potential risk that the proposed legalisation would pose to ‘immature persons’.^75^ McQuaid’s view that ‘any such contraceptive act is always wrong in itself’ went beyond the papal encyclical *Humanae Vitae* of 1968, which had declared that natural methods of family planning might be permissible.

Some women had more difficulties with reconciling their family planning choices with their Catholic faith. In a response to a piece by an Irish priest on the theme of contraception in the *Irish Times* in 1970, one woman explained about the personal conflict she felt in using contraception:

Many times I have been present at Mass in misery, staying away from Holy Communion and worrying about the bad example shown to my older children. I feel in my heart that it can’t possibly offend God to show love for one’s husband while at the same time trying to prevent conception, but after years of strict Irish Catholic upbringing, scruples are hard to overcome.^76^

Through disobeying the papal teachings on contraception, this particular woman felt significant emotional distress and guilt, and while she tried to rationalise her birth control practices, she felt that her life following Catholic teachings made it difficult to avoid these feelings. Others were unable to take the contraceptive pill for health reasons and wrote of their difficulties in attempting to use the rhythm method. Writing to Archbishop John Charles McQuaid in 1971, one woman explained:

The rhythm method does not work for me and speaking as a trained nurse I assure you we made no mistakes. We tried total abstinence but my husband does not agree with this and says this is not marriage and that if he had wanted to lead a celibate life he would have chosen it. Have you any idea of the mental and spiritual anguish of a Catholic mother placed in this predicament!! And being told by Drs that I cannot use oral contraceptive methods?^77^

This woman’s account illustrates her anguish at trying to avoid pregnancy but also maintain marital harmony. Unfortunately we do not know McQuaid’s response. Other women went to their local priests for advice on the matter. Maura, interviewed by *Woman’s Way* in 1973, reported that her local priest was sympathetic to her personal dilemma about taking the contraceptive pill. Although he advised that contraception was against Church teachings, he explained that ‘he personally believed that it was a matter for her own conscience’. However, this may not have been a typical experience. Angela, the mother of four, went to her priest for advice and had a markedly different experience: ‘He was furious. He gave me a lecture about the evilness of contraception and how I would be flaunting [sic] the authority of the Holy Father.’^78^ June Levine, a member of the Irish Women’s Liberation Movement, and a journalist for Irish women’s magazine *Creation*, wrote about a letter she received from a woman on the topic of contraception in the 1960s:

‘I asked the priest if I could use the pill’, a woman told me, ‘and he said no, so I’m off it again. I dread it, and then what when this baby arrives?’. Her letter stunned me on two counts, knocked me from side to side. I couldn’t identify with her taking the priest’s word for her sex life. How could such a morbid approach to sexuality be embraced in spiritual terms? And she’d said ‘I’m off it again’, as if she hadn’t anything to do with the whole business.^79^

As Hilliard has suggested, many women in her study had a dread of going to confession and were faced with the choice ‘to stop avoiding further pregnancies or be refused absolution’.^80^ Some priests were sympathetic.^81^ It is also possible that information on sympathetic priests was also circulated by word-of-mouth among women. Nuala Fennell wrote in her 2009 memoir that:

Similarly there was a small network of understanding priests to whom to confess. A Dublin acquaintance of mine who was on the pill for years, travelled the three hundred and twenty-mile round trip every month to confess to a priest in Cork.^82^

Some of the interviewees in Leane’s study also reported a priest in Cork city who was willing to provide absolution to women taking the contraceptive pill.^83^ Similarly, as Diane Gervais and Danielle Gauvreau found in their study of family limitation in Quebec 1940–70, some women in Quebec ‘shopped around’ for an understanding priest who would not refuse them absolution at confession, while Leslie Tentler has shown similar practices in the United States.^84^ Frank Crummey, family planning activist and a founding member of family planning clinic, Family Planning Services, believed that many Irish women simply did not tell their priests about their decision to use artificial contraception. Interviewed in 1979 for Rosita Sweetman’s book, *On Our Backs*, he stated:

But this business about artificial contraception being a mortal sin, I think the women just don’t tell the priest anymore. I mean are the 30 000 people on our mailing list all non-Catholics? And what about the 70 000 Irish women on the Pill, are they all non-Catholics? And if they’re just using the Pill as a cycle regulator then we must have the unhealthiest women in the world.^85^

As Rusterholz has found in her study of contraceptive practices in Switzerland from 1955 to 1970 ‘for those who were aware of the Catholic Church’s condemnation of birth control, their adherence to this doctrine was not strong enough to prevent them from using birth control’. The majority of the 75% of respondents to her study who confirmed they were practising Catholics were using forms of contraception banned by the Catholic Church.^86^ The decision to take the pill therefore often came down to personal conscience, and the contemporary literature suggests that many Irish women believed that decisions around family planning were a matter for themselves. In an article in 1966 in *Woman’s Way* magazine, Monica McEnroy explained that she believed that the majority of women in Ireland ‘consider contraception a matter for people themselves. These women feel that they should be able to walk into their hospital clinic, dispensary or private doctor and be investigated for a suitable ovulation-suppressant (Pill), or some other effective form of conception control, if necessary, without any fuss, publicity or special permissions. These women believe that they and they alone know what is necessary for the physical, mental and moral welfare of themselves and their families’.^87^ In 1964, Máire Mullarney asked her doctor for the contraceptive pill. Mullarney, a Catholic, had read a report by Father Bernard Haring ‘that it might be possible for a mother who was not able to breastfeed to take the Pill to prevent conception, on the grounds that this would have been prevented by lactation’. Mullarney had a baby who was five months old and she wasn’t breast-feeding: ‘I asked our doctor for the Pill right away. I did not consult anyone with a Roman collar. I might not have picked one who read the more adventurous journals.’^88^

It is clear that the contraceptive pill became an important symbol of family planning in debates around the legalisation of contraception. While there has been some research conducted on the role of politicians and the medical profession in debates around family planning, less attention has been paid to the significance of anti-contraception campaigners and the importance of feminist groups to these debates in the mid-1970s.^89^ Through an exploration of the literature of these groups and oral history interviews with former members, I seek to show how the contraceptive pill became an important focus of their campaigns. While both groups had differing views about the legalisation of contraception, the side effects of the contraceptive pill was an important area of focus for members of both sides of the debate.

## 3 The Irish Women’s Movement and the Pill

In this section I seek to explore the stance of Irish feminist groups on the contraceptive pill in the 1970s. The three groups I will focus on here include the IWLM, the short-lived Women’s Liberation Movement and IWU and their offshoot, CAP. While there have been important studies of these groups, less attention has been paid to their involvement in debates around the contraceptive pill.^90^ As will become clear from the discussion below, each of these groups were concerned with the fact that, due to lack of availability of other options, many Irish women were taking the contraceptive pill regardless of its potential health risks. In the United States, in the late 1960s and early 1970s, the perceived over-prescription of the contraceptive pill was critiqued by feminist activists who believed that women’s reproductive healthcare had become over-medicalised and were concerned with the side effects on women’s health.^91^ Following the publication of Barbara Seaman’s book, *The Doctor’s Case Against the Pill*, feminist campaigners began to ‘vocalise the shared perception that the medical profession was condescending, paternalistic, judgemental and non-informative’.^92^ And, as Elizabeth Siegel Watkins has asserted, ‘the feminist critique of medicine grew from several sources of dissatisfaction, but the controversy over the safety of the pill and the importance of informed consent in its use served as a catalyst for the growth of the women’s health movement’.^93^ Fundamentally, members of the women’s movement resisted viewing the contraceptive pill as a positive boon for women and moved towards emphasising the importance of choice with regard to contraceptive methods. While some feminists emphasised that women-centred contraception was positive in that it provided women with control over their reproductive lives, they recognised that this often meant that the responsibility for birth control lay with women.^94^ Others encouraged more research into male methods and for male partners to share more of the burden.

The IWLM was the first Irish women’s group to take a stand on the government’s laws on contraception. The group lasted just over a year, but had a significant impact. Through a variety of tactics, including walk-outs of Catholic masses, protests at government buildings, the group directly took on the Catholic hierarchy and Irish government. Although the IWLM was short-lived, it had an important impact on the Irish public because of its prominence in the Irish media.^95^ A largely middle-class group of Irish women including left-wing activists, trade unionists, journalists and housewives had formed the IWLM in 1970.^96^ Several of the founder members of the IWLM held prominent positions as journalists, while others had backgrounds in left-wing and republican politics. This meant, in Yvonne Galligan’s view, that ‘the small group had a considerable reservoir to draw on when seeking to disseminate feminist ideas and information in a country still quite insular in its social perspectives’.^97^ Contraception was an important focus for the IWLM. The Irish laws against family planning were critiqued in a section of their 1971 booklet *Irishwomen: Chains or Change* entitled ‘Incidental facts’, which also noted the lack of childcare facilities, playgrounds and creches, baby-minding regulations, the option to divorce, and re-training facilities for women. The section on the family planning laws drew attention to the hypocrisy of the law in that it was possible for Irish women to get access to the contraceptive pill as a ‘cycle regulator’ and reported that ‘25 000 Irishwomen use it, ostensibly under the guise of a medicine to regularise the menstrual cycle’. Founder member, Mary Kenny, recalled conversations around the pill at IWLM meetings, ‘in which it was suggested that perhaps the Pill was too widely used – because there were not sufficient alternatives’.^98^ As Dr Eimir Philbin Bowman, a doctor and member of the Irish Women’s Liberation Movement, explained in an oral history interview, for many users, the benefits of the contraceptive pill outweighed the potential health risks:

The notion that you could pop something into your mouth, the convenience of it, the fact that you could do it privately. There were quite a number of women who were on the pill whose husbands probably didn’t know they were on the pill. That it didn’t interfere with sexual intercourse and the woman was looking after her own fertility, but nevertheless you could say that women were taking all the risks that went with the early pill. But that obviously was, to them, much more acceptable than the risk of having sequential babies.^99^

The IWLM conducted a number of direct action activities, culminating in their Contraceptive Train stunt on 22 May 1971. Forty-seven members of the IWLM boarded the 8am train from Dublin to Belfast with the aim of purchasing contraceptives in the north and travelling back with them in order to highlight the hypocrisy of Irish law.^100^ The women returned to Dublin to face customs officers, who allowed them to retain their purchases and did not arrest them. Members of the IWLM were concerned by the fact that the contraceptive pill was often prescribed as a cycle regulator to Irish women, despite the fact that this may not have been the most suitable contraceptive for them, and sometimes produced side effects. Other contraceptives, such as the diaphragm, condoms and the coil, were available to middle-class women through a legal loophole from family planning clinics in Dublin or to women who were able to travel to the UK to obtain them. In a statement read by founder member Nell McCafferty following the event, the Irish government was accused of ‘criminal irresponsibility’ in permitting 26 000 women to use only the contraceptive pill because that was the only contraceptive available to them, despite the fact that the pill was in many cases ‘medically unsuited and damaging to the woman who might otherwise, in all conscience, choose other methods at present illegal’.^101^

Following the disbandment of the IWLM, members of the women’s movement split into other groups, including the short-lived Women’s Liberation Movement, which published a magazine called the *Fownes Street Journal*. Articles in this journal emphasised the potential side effects of the contraceptive pill. According to one article in a 1973 issue of the magazine:

Now obviously the ‘pill’ has been a boon to many women whose married lives have been affected by fear of further pregnancies but it is wrong that it should be the only ‘legal’ means, when some are unable to take it and it has not been in use for long enough to fully assess its side-effects.^102^

Moreover, the author argued that the fact that the ‘female contraceptive pill is accepted provided its real use is concealed, while vasectomy which seems an eminently suitable arrangement for married men of reasonable age who are parents of a reasonable number of children, is not’ was ‘typical of the male-dominated society in Ireland and the general hypocritical attitudes prevalent here’.^103^ Similarly, in a later issue of the magazine that year, Norah Kelly questioned why most forms of contraception were female-centred, suggesting that ‘the great relief that women have experienced as a result of the introduction of more freely available methods should not blind them to the fact that it is still a very one-sided affair’. Kelly encouraged women to press for more research into other contraceptive methods, ‘or at least make sure that the lag in the burden-sharing is due to genuine technical difficulty, rather than the desire not to come between a man and his comfort’.^104^

It was not until the foundation of IWU in 1975, that there was ‘a women’s liberation group of any comparable scale to the IWLM’.^105^ Although the group used similar tactics to the IWLM, such as direct action and consciousness raising, IWU was arguably more politicised.^106^ Contraception was a key mobilising issue for this group. At a Contraception Workshop held by the group in 1975, it was agreed that if women were given control of their bodies through access to contraception, it would be possible for them to gain more freedom and choice in relation to employment opportunities. In addition, the demand for contraception was linked to ‘the right of all women to a self-determined sexuality’.^107^ The CAP, established in spring 1976, emerged from the IWU Contraception Workshop.^108^ The organisation also included members from other interested groups. For instance, at a meeting of the CAP in June 1976 at Buswell’s Hotel in Dublin, attendees included members of Women’s Aid, the IFPA, North Dublin Social Workers, Women’s Liberation Movement, Women’s Progressive/Political Association, Family Planning Services, the Labour Women’s National Council and Irishwomen United, all of whom were women, with the exception of Robin Cochran, representative of Family Planning Services.^109^ However, it was predominantly members of IWU who were the driving force behind the campaign, which included the setting up of a shop, Contraceptives Unlimited, to illegally sell non-medical contraceptives in 1976. Discussions around the contraceptive pill by IWU moved to a rhetoric which focused on class and geographic disparities, women’s entitlement to a choice of contraceptives and an emphasis on the side effects of the pill.

A statement issued by IWU in 1976 outlines their key demands regarding contraception and their concerns about the contraceptive pill:

Contraception is central to our struggle as women. It is a minimum right. It is the first step towards a fuller control of our lives. We do not demand population control when THEY decide how many children WE should have. We demand real control where we decide how many children we want. Therefore: We demand the BEST and SAFEST forms of contraceptives FREE. Women are not guinea-pigs. We don’t want to have to put up with expensive contraceptives that either don’t work or make us feel ill or depressed.^110^

Fundamentally, for members of IWU, it was important that women had a range of options regarding contraception, but that doctors should play more of a role in listening to their patients and discussing the options. In an article in *Banshee* magazine in 1976, the group wrote:

But there is more to birth control than swallowing a pill, or inserting a device. Contraception is as much a sociological issue as it is medical. It should not be treated like codeine, available only through chemists shops, as though that were the only safeguard required.^111^

Similarly, the Women’s Liberation Movement in England, ‘accused physicians of prescribing the Pill carelessly, without examining their patients or asking about their former experiences’. IWU members were concerned with the fact that many Irish women were using the contraceptive pill when it was not suitable for them, but they also expressed concern that doctors did not fully inform patients of the pill’s side effects.

According to Roisin Boyd, a member of IWU writing in 1977:

Because of this lack [of legal contraception], many women are using the Pill when it is unsuitable for them. Also the unavailability of contraception in country areas, means that women are dependent on sympathetic doctors or chemists. The situation at the moment is intolerable. There is an attitude prevalent among many doctors that you’re lucky to be getting any contraception at all and they are reluctant to advise women on which is the best available method. Some doctors are still prescribing Pills with an 80% oestrogen content and neglecting to inform women that they might suffer side-effects such as thrush if using the Pill at all.^112^

The key aim for IWU members therefore was that women should ultimately have a range of options and that doctors should be equipped to provide these. As Anne Speed (IWU/CAP) explained to me in an oral history interview:

In relation to contraception, yes there were women going for the pill as cycle regulator. But really they should’ve been getting advice for forms of contraception and other things that were suitable.^113^

Similarly, as Ruth Torode (IWU/CAP) explained to me, while it was ‘assumed that the pill was the easiest for women to use’, some women had difficulty with it, and IWU’s demands focused on ‘choice and information and care’.^114^ Articles published by IWU members in feminist magazines in the 1970s tended to focus on the potential side effects of the contraceptive pill. Writing in *Banshee* in 1977, for instance, one woman emphasised that the pill had led to increased weight gain and her concerns about the hormones of the pill:

Unfortunately though, the next year saw a rapid expansion in my measurements due in part to the pill. My mother when she saw me was horrified at her enormous daughter but she took it well – better fat than pregnant I suppose…after five years on and off various pills its novelty was wearing off though not its effects. Meanwhile my lifelong interest in food was leading me into an interest in whole foods and vegetarianism. This was accompanied by a general interest in my physical well being. What I saw was an inconsistency between such a way of life and the fact that I allowed my whole hormonal system to be regulated by a little white pill. But what alternative?’^115^

This quote is revealing – for this particular woman’s mother, the side effects of the pill were a small price to pay in return for safety from pregnancy. However, as the woman became more interested in her physical well-being and alternative diets, she became concerned with the hormones of the pill and potential side effects. Yet, her testimony highlights that there wasn’t any alternative available to her. Similarly, Ann O’Brien, an IWU member, writing in feminist magazine *Wicca* in 1977 explained:

I was on the Pill for 2 years and generally speaking I found it alright. It was great not to have interruptions or worries, but even though I know a certain amount about how the Pill works, it worried me if I missed one or two. I didn’t know what was happening inside. Also, of course, I was conscious that I was taking a pill every day that may be affecting other parts of my body and I didn’t know how. ^116^

Similarly, another woman described her experience on the contraceptive pill as follows:

In the early days of contraception (which then as now mainly meant the pill) sympathetic doctors outside the Family Planning Clinic in Merrion Square would prescribe it without much knowledge of dosages. At least, they never asked me anything about my health, periods or took my blood pressure. That’s why I almost blacked out one day, when I was driving a motor bike round Stephens Green. It was 1964 and that particular high dose pill was later withdrawn. Years later I found a combination of oestrogen and progesterone to suit me and I used it successfully until I stopped to get pregnant…^117^

This woman’s testimony suggests that her doctor too readily prescribed her the pill without consideration of the dosage or side effects. Moreover, she condemned the emphasis on women’s responsibility for contraception, suggesting that ‘a male pill would be simpler on men’s physiology since women’s hormones are more complicated. But drug companies know they will make more from women and have held up research into a male pill. In the interim women must educate men that contraception is their business too’.^118^

## 4 The Irish Family League

The Irish Family League (IFL) was a group of Catholic campaigners founded in May 1973 and one of the most prominent organisations that campaigned against the legalisation of contraception. The league had been formed following a visit to Ireland by American pro-life campaigner Father Paul Marx, and because of the press campaign for the liberalisation of the contraceptive law.^119^ Its key aims, summed up in their publication *Is Contraception the Answer?*, centred around maintaining articles of the Irish constitution which enshrined Christian values and ensuring the continuing existence of laws which enshrined Christian values, and opposing permissive legislation. The group was opposed to the legalisation of contraception, divorce, abortion and euthanasia, and aimed to combat pornography, violence and secularism. They believed in the promotion of the Christian education of young people and aimed to promote the welfare of the family.^120^ The key figure involved in the IFL was John O’Reilly, who went on to be heavily involved in Ireland’s Pro-Life Amendment Campaign in the 1980s, and Mary Kennedy, who acted as secretary to the group. The executive was composed of fewer than ten individuals, who met on a weekly basis. The IFL was extremely active in writing to the press and lobbying politicians; according to an interview with Mary Kennedy, by 1980 they had 2000 members.^121^ The IFL had a number of arguments against the legalisation of contraception, which I will outline here. For the purposes of this article, I will focus on the IFL’s arguments that centred on the contraceptive pill. The group argued that this contraceptive posed health risks and was also a form of abortifacient.

The IFL’s first major concern centred on the idea of ‘abortion by contraception’ in that they believed that certain forms of contraceptives, notably the contraceptive pill and the IUD, were forms of abortifacients.^122^ This stemmed from the belief that life began at the moment of conception and that, therefore, a contraceptive that prevented a fertilised ovum from implanting was a form of abortion. In their publication *Is Contraception the Answer?*, the group argued that ‘many brands of the “pill” may be, and the Intra-Uterine Device certainly is, ABORTIFACIENT: i.e. instead of preventing conception, their action is to abort the fruits of conception at an early stage’.^123^ While the publication explained that the early form of the pill was purely contraceptive in action, the newer low-dosage pills were ‘less successful in suppressing ovulation, but if they fail to do this, they will abort the fertilised ovum as does the IUD’.^124^ By 1976, the group’s language around this issue had become more pro-life in nature. In their publication *Why You Should Oppose Contraception*, they explained ‘many so-called contraceptives are in fact abortifacients and human life is so precious that nobody has the right to kill’.^125^

The group’s publications and letters to newspapers were rigorously researched and often included references to medical texts which were used to back up their arguments. In *Is Contraception the Answer?*, the group argued that ‘apart from their being morally impermissible, the most popular, i.e. the most reliable, so-called contraceptives are also serious medical risks’. This publication drew attention to allegations that IUDs had potential carcinogenic effects, while also asserting ‘the pill is considered so dangerous that one of the largest US brokerage companies providing insurance cover for doctors has advised all its clients to obtain signed statements from patients acknowledging that they have requested birth-prevention pills despite their awareness of the serious risks involved’.^126^ A copy of the form was included in the publication as an appendix. In a letter to the *Irish Times* in 1974, Mary Kennedy asked:

Is it really a human right to dose women with pills strong enough to prevent a natural function, or to insert in them devices which even doctors of the Family Planning Clinic admit they do not know exactly how they work?^127^

The language utilised by the IFL was often foreboding. In a 1976 letter to the *Irish Times*, Mary Kennedy referred to a recent editorial of the *British Medical Journal* on the side effects of the contraceptive pill, summing its conclusion up as ‘the statement that those women who use them must be prepared to pay the price’.^128^ Such letters did not go unnoticed and often sparked debate in the letters pages of the *Irish Times*. In 1977, Janet Farrar, from Wexford, for instance, replied to Kennedy’s letter concerning the side effects of the pill

As for her claim that the Pill and the IUD are abortifacient, and her undated and probably distorted citing of the *British Medical Journal*, I am sure any impartial doctor would agree that she is grossly exaggerating minimal side-effects. Any method of birth control involves a small possibility of unwanted effects (as does using a cooker or crossing the road), but with proper medical advice and checking it can be still further reduced. And these minimal side-effects are a drop in the ocean compared with the psychological dangers of the rhythm method (leaving aside its unreliability).^129^

Kennedy replied a few days later with more statistics relating to the side effects of the contraceptive pill. She referred to ‘the four deaths here in the past year as a direct result of the Pill’ and the fact that Irish family planning clinics had recently withdrawn two brands of contraceptive pills which they had been providing for several years due to ‘adverse reports from America’.^130^ The thalidomide disaster of the early 1960s was often used by the group in discussions around the contraceptive pill. In their publication *Alert: Oral Contraceptive*, the IFL outlined the potential health risks of the contraceptive pill. The publication argued that there were parallels between the contraceptive pill and the thalidomide drug, suggesting that the contraceptive pill, like thalidomide, had been inadequately tested before it became available on prescription to women, and the publication drew attention to accounts by American physicians concerning the dangers of the pill.^131^ Writing to the *Irish Times* in 1975, Mary Kennedy referred to the American Food and Drugs Administration (FDA) report on the contraceptive pill, and also commented that ‘the FDA were first to warn of the Thalidomide drug’.^132^ The following year, in another letter, she asked: ‘Have we so soon forgotten the thalidomide children and the tragedy of their lives?’^133^ Kennedy also alleged that the contraceptive pill might have potential long-term effects on the third and fourth generation of users of chemical contraception, and cited the work of German doctor Siegfried Ernst while also commenting that ‘genetic damage has also been noted in the USA’.^134^ In her view, if groups such as the CAP were successful in having contraceptives made available, the taxpayer would not only have to pay for the provision of the services but would also ‘have to provide compensation when the users suffer damage to their health’.^135^

The IFL perceived that family planning groups were concerned with the profits to be made from artificial methods of birth control, and therefore did not advocate natural methods of family planning. They often drew attention to the commercial concerns relating to the prescription of the contraceptive pill. In a 1976 letter to the *Irish Times*, Mary Kennedy asserted that pills and devices were being ‘pushed in this country by concerns whose motivation is purely commercial’ and that such concerns were being supported by family planning groups, ‘the young people in Irish Women United and by some in the universities’. This meant, in her view, that all publicity was being given to artificial methods and none to natural methods. ^136^ In addition, Kennedy believed that Irish doctors prioritised prescribing the pill over other forms of contraception:

We have been told by women seeking information on natural methods: ‘He would not spend five minutes to discuss the problem with me, but just wanted to write a prescription for the Pill.’ Other women have told us that doctors did their best to persuade them to take the Pill when they went for a post-natal check-up, even though these women had not asked for advice and were indeed already adequately spacing their families.^137^

In Kennedy’s view, prescribing the contraceptive pill meant that doctors did not have to spend time advising on other methods which would take more explanation. Furthermore, in a letter later that year, Kennedy asserted that she believed there was opposition towards natural methods such as the Billings method because ‘there is no money to be made from showing a woman how to learn and use this method’ and ‘because it is permissible for Catholic women to use it, to space their families, it is not acceptable to those who hate the Catholic Church and her teaching’.^138^ She also claimed in 1978 that the example of the ‘married woman with 10 children and a drunken husband has been dropped in favour of contraceptives for the young and single’ because the latter would ‘provide a more lucrative market for the trade’.^139^

In addition to these arguments against the contraceptive pill, the group also expressed its fears about the ‘corruption of youth’ – that the introduction of contraception would lead to an increase in promiscuity among adolescents. The IFL was also concerned that the introduction of contraception would lead to an increase in rates of illegitimacy and venereal disease. Britain was also referred to in many of their publications as an example of a permissive society and the effects from legalisation of contraception. IFL members were concerned with the rates of venereal disease, illegitimacy and abortion in Britain and these statistics were often cited in their publications. Britain was depicted as a permissive society because contraception and abortion were legal there and there were high rates of teenage pregnancy. According to Mary Kennedy, in a letter to the *Irish Press* in 1975, ‘In England, abortion has debased the profession of medicine and of nursing to that of paid killer, highly profitable to those involved’. Kennedy argued that this ‘must be an example to us of what can happen when the selfishness of the contraceptive society is given free rein’.^140^ However, the key concern for members of the IFL, and indeed, for many members of the Irish public who were against contraception, was the notion that if contraception was introduced, other liberal reforms, such as divorce and, in particular, abortion, would soon follow.

## 5 Conclusion

By 1979, when contraception was legalised in Ireland, albeit for *bona fide* family purposes only, debates surrounding the pill continued, and members of the women’s movement began to focus on non-hormonal alternatives. Róisín Conroy, a member of IWU, explained in 1979:

The debate about the dangers of the ‘Pill’ goes on, with many pros and cons from doctors and little decisive information for women to go on. Many women’s bodies reject the intrauterine devices and the most effective, larger varieties are not suitable for women who have never borne children. Whatever the real story about the ‘Pill’, its failures and its side effects, the fact remains that a good many women don’t trust it and have gone back to that antique rubber mechanism, the diaphragm with cream/jelly. They know it does nothing to their body chemistry, it lasts for quite a while, and there is a curious psychological advantage (commonly considered a drawback) to the fact that they must exercise choice each time they use it.^141^

Conroy encouraged research into new and better techniques of contraception. After legalisation, women’s magazines also began to focus on publicising non-hormonal methods of contraception with articles on contraceptives moving away from the previous focus on the contraceptive pill. A report by the Irish Medical Association into the side effects of the contraceptive pill, published in the year before legalisation, was widely publicised and also helped to bolster fears surrounding the side effects of the pill.^142^

Evidently, debates around the contraceptive pill in 1960s and 1970s Ireland were complex. As in England, the contraceptive pill became a synonym for contraception more generally, and a means for the press to discuss the issue. The fact that some women could obtain the contraceptive pill through lying about their menstrual difficulties, illustrates the significant hypocrisy of the Irish ban on contraception, and the contraceptive pill represented a symbol of class disparities with regard to access to contraception pre-legalisation. Women who took the contraceptive pill evidently displayed significant agency in gaining access to it, and the circulation of knowledge regarding how to get access, and from whom, shows the importance of women’s networks in helping women to circumvent the legislation. However, as some accounts illustrate, the decision to take the contraceptive pill could sometimes result in a dilemma with regard to conscience for Catholic women; however, such women often justified taking the contraceptive pill for economic factors. Finally, it is evident that the contraceptive pill became an important emblem of the debate around Irish family planning laws for both campaigners in favour of changes to the legislation, and campaigners against. For anti-contraception campaigners such as the Irish Family League, a focus on the pill as an ‘abortifacient’ and the discussion of its potential side effects, helped to assert their stance on contraception, but also meant that discussions of contraception could not be separated from discussions of abortion. For members of the Irish women’s movement, given the potential side effects of the pill and its lack of suitability for some women, it was essential that women should be given a range of options with regard to contraception. They also criticised the focus on female-centred contraceptives which had led to the responsibility for contraception being placed on women. In this regard, the pill became an important emblem of Irish women’s lack of choice in access to contraception as well as highlighting the significant class and geographic disparities. Following legalisation, access remained restrictive, however, now that a range of contraceptive options were in theory, legally available to women, the previous focus on the pill in the Irish media shifted to non-hormonal methods of contraception.

## Footnotes

1.
Dorine Rohan, *Marriage Irish Style* (Cork: Mercier Press, 1969). A significant portion of the book was first published in the *Irish Times* newspaper, and was described at the time as ‘provocative and probing’. Diarmaid Ferriter, *Occasions of Sin: Sex and Society in Modern Ireland* (London: Profile Books, 2010), 336.

2.
Rohan, *Ibid.*, 97.

3.
Rohan, *op. cit.* (note 1), 97.

4.

*Woman’s Way*, 28 March 1969, 10-11.

5.
Similarly, as Caroline Rusterholz’s study of contraceptive practices in Switzerland 1955–1970, has shown, some Catholic women justified their use of birth control for economic reasons. Caroline Rusterholz, ‘Reproductive Behavior and Contraceptive Practices in Comparative Perspective, Switzerland (1955–1970)’, *The History of the Family*, 20, 1 (2015), 41–68, on 51.

6.
This article will focus on the Republic of Ireland, or the twenty-six counties, excluding Northern Ireland. For important studies of this legislation see: Sandra McAvoy, ‘Its effect on public morality is vicious in the extreme: defining birth control as obscene and unethical, 1926–32’, in Elaine Farrell (ed.), *She Said She Was in the Family Way: Pregnancy and Infancy in Modern Ireland* (London: Institute of Historical Research, 2012); Sandra McAvoy, ‘A perpetual nightmare. Women, fertility control and the Irish state: the 1935 ban on contraceptives’, in Margaret Preston and Margaret Ó hÓgartaigh (eds), *Gender and Medicine in Ireland 1700–1950* (Syracuse: Syracuse University Press, 2012); Chrystel Hug, *The Politics of Sexual Morality in Ireland* (Basingstoke: Palgrave Macmillan, 1999), 79–82. For a useful overview, see: Anne Speed, ‘The struggle for reproductive rights: a brief history in its political context’, in Ailbhe Smyth (ed.), *The Abortion Papers* (Dublin: Attic Press, 1992), 85–98.

7.
McAvoy, ‘A perpetual nightmare’, *Ibid.*, 202.

8.
See: Diarmaid Ferriter’s *Occasions of Sin: Sex and Society in Modern Ireland* (London: Profile Books, 2009). For further discussion of the debates surrounding the Health Family Planning Act, see: Aidan Beatty, ‘Irish Modernity and the Politics of Contraception, 1979–1993’, *New Hibernia Review*, 17, 3 (2013), 100–18; and Brian Girvin, ‘An Irish Solution to an Irish Problem: Catholicism, Contraception and Change, 1922–1979’, *Contemporary European History*, 27, March (2018), 1–22. On the role of the medical profession and the Fertility Guidance Company, see: Deirdre Foley, ‘“Too Many Children?”: Family Planning and *Humanae Vitae* in Dublin, 1960–1972’, *Irish Economic and Social History*, 46, 1 (2019), 142–60; and Mary E. Daly, *Sixties Ireland: Reshaping the Economy, State and Society, 1957–73* (Cambridge: Cambridge University Press, 2016), 148–50.

9.
Lindsey Earner-Byrne, ‘Moral prescription: the Irish medical profession, the Roman Catholic Church and the prohibition of birth control in twentieth-century Ireland’, in Catherine Cox and Maria Luddy (eds), *Cultures of Care in Irish Medical History, 1750–1950* (Basingstoke: Palgrave Macmillan, 2010).

10.
See: Lindsey Earner-Byrne and Diane Urquhart, *The Irish Abortion Journey, 1920–2018* (Basingstoke: Palgrave Macmillan, 2019), 51–68; Leanne McCormick, ‘“The Scarlet Woman in Person”: The Establishment of a Family Planning Service in Northern Ireland, 1950–1974’, *Social History of Medicine,* xxi, 2 (2008), 345–60; Leanne McCormick, *Regulating Sexuality: Women in Twentieth-century Northern Ireland* (Manchester: Manchester University Press, 2009); Greta Jones, ‘Marie Stopes in Ireland: The Mother’s Clinic in Belfast, 1936–47’, *Social History of Medicine*, 5, 2 (1992), 255–77.10.1093/shm/5.2.25511623088

11.
Hug, *op. cit.* (note 6), particularly chapters 3 and 4; Emilie Cloatre and Máiréad Enright,‘“On the Perimeter of the Lawful”: Enduring Illegality in the Irish Family Planning Movement, 1972–1985’, *Journal of Law and Society*, 44, 4 (2017), 471–500; Emilie Cloatre and Máiréad Enright, ‘McGee V Attorney General’, in Máiréad Enright, Julie McCandless and Aoife O’Donoghue (eds), *Northern/Irish Feminist Judgments: Judges’ Troubles and the Gendered Politics of Identity* (Oxford: Hart Publishing, 2017), 95–108. Linda Connolly, *The Irish Women’s Movement: From Revolution to Devolution* (Basingstoke: Palgrave Macmillan, 2002); Linda Connolly and Tina O’Toole, *Documenting Irish Feminisms*, (Dublin: Woodfield Press, 2005).10.1111/jols.12055PMC575188529307949

12.
See: Lindsey Earner-Byrne and Diane Urquhart, *op. cit.* (note 10); Ann Rossiter, *Ireland’s Hidden Diaspora: The Abortion Trail and the Making of a London-Irish Underground, 1980–2000* (London: IASC Publishing, 2009). For important studies of abortion in Ireland in the early twentieth century, see Cara Delay, ‘Pills, Potions, and Purgatives: Women and Abortion Methods in Ireland, 1900–1950’, *Women’s History Review*, 28, 3 (2019), 479–99 and ‘Kitchens and Kettles: Domestic Spaces, Ordinary Things, and Female Networks in Irish Abortion History, 1922–1949’, *Journal of Women’s History*, 30, 4, (2018), 11–34.

13.
Emilie Cloatre and Máiréad Enright, *op. cit.* (note 11), 471–500, on 475. As Cloatre and Enright point out, more attention has been given to government and medical debates surrounding family planning than the role of activists groups.

14.

Betty Hilliard , ‘The Catholic Church and Married Women’s Sexuality: Habitus Change in Late 20th Century Ireland’, Irish Journal of Sociology, 12, 2 (2003), 28–49 and Máire Leane, ‘Embodied sexualities: exploring accounts of Irish women’s sexual knowledge and sexual experiences, 1920–1970’, in Máire Leane and Elizabeth Kiely (eds), *Sexualities and Irish Society: a Reader* (Dublin: Orpen, 2014), 29–56.

15.
See, for instance, Teresa Ortiz-Gómez and Agata Ignaciuk, ‘“Pregnancy and Labour Cause More Deaths than Oral Contraceptives”: The Debate on the Pill in the Spanish Press in the 1960s and 1970s’, *Public Understanding of Science*, 24, 6 (2015); Agata Ignaciuk, ‘Paradox of the pill: oral contraceptives in Spain and Poland (1960s–1970s)’, in Ann-Katrin Gembries, Theresia Theuke and Isabel Heinemann (eds), *Children by Choice?* (De Gruyter, 2018), 95–111. Tiago Pires Marques, ‘The politics of Catholic medicine: “the pill” and *Humanae Vitae* in Portugal’, in Alana Harris (ed.), *The Schism of ’68: Catholicism, Contraception and* Humanae Vitae *in Europe, 1945–75* (Basingstoke: Palgrave Macmillan, 2018), 161–86. For a broad history of the contraceptive pill see Lara Marks, *Sexual Chemistry: A History of the Contraceptive Pill* (New Haven, CT: Yale University Press, 2010).10.1177/096366251350976424259515

16.
This is part of a wider research project which utilises oral history research in order to illuminate women and men’s personal experiences. This research is ongoing; however, some extracts from oral history interviews that have been conducted to date are included here. These include interviews with members of the family planning movement, campaigners both for and against legalisation, as well as interviews with men and women born between 1925 and 1955. The remaining oral history research will further illuminate family planning practices of Irish couples in the 1960s, 1970s and 1980s.

17.
For a recent study of women’s magazines in Ireland, see Caitriona Clear, *Women’s Voices in Ireland: Women’s Magazines in the 1950s and 1960s* (London: Bloomsbury, 2016), at 46–8. *Woman’s Way* magazine appeared every fortnight from 1963 until August 1966, when it began to be published weekly. The magazine was aimed at women of all ages and covered a range of themes, from cookery to fashion to current affairs. See: Clear, *ibid.*, 65.

18.
For instance, as the author of a letter to the *Irish Times*in 1970 explained, ‘Because of the ban on information, the “pill” has become synonymous with contraception in this country. Diaphragm seems to be a dirty word and the IUD unheard of: Contraception: what do you think?’, *Irish Times*, 22 December 1970, 6.

19.

Mary E. Daly , Sixties Ireland: Reshaping the Economy, State and Society, 1957–1973 (Cambridge: Cambridge University Press, 2016), 146.

20.
Ortiz-Gómez and Ignaciuk, *op. cit.* (note 15), 660.

21.
Advertisement for Lyndiol 2.5, *Journal of the Irish Medical Association*, LX, 365 (November 1967), no page number.

22.

Elizabeth Siegel Watkins , On the Pill: A Social History of Oral Contraceptives, 1950–70 (Baltimore: Johns Hopkins University Press, 1998), 2.

23.

Hera Cook , The Long Sexual Revolution: English Women, Sex and Contraception: 1800–1975 (Oxford: Oxford University Press, 2004), 272.

24.

Eva-Maria Silies , ‘Taking the Pill After the “Sexual Revolution”: Female Contraceptive Decisions in England and West Germany in the 1970s’, European Review of History: Revue européenne d’histoire, 22, 1 (2015), 41–59, on 43.

25.

Alex Mold , Making the Patient-consumer: Patient Organisations and Health Consumerism in Britain (Manchester: Manchester University Press, 2016), 18–19.

26.
Cook, *op. cit.* (note 23), 287–91.

27.
Cook, *op. cit.* (note 23), 287–91.

28.
‘The pill in Ireland: a short review of the facts’, *Irish Times*, 1 August 1968, 6.

29.
Mary Maher, ‘A short history of the pill in Ireland’, *Irish Times*, 14 March 1968, 8.

30.
Cook, *op. cit.* (note 23), 290.

31.
Oral history interview with Professor Dermot Hourihane, 04-01-2017.

32.
Watkins, *op. cit.* (note 22), 54.

33.

Andrea Tone , ‘Medicalizing Reproduction: The Pill and Home Pregnancy Tests’, Journal of Sex Research, 49, 4 (2012), 319–327, on 321.2272082310.1080/00224499.2012.688226

34.
Ursula Barry, ‘The movement, change and reaction: the struggle over reproductive rights in Ireland’, in Ailbhe Smyth (ed.), *The Abortion Papers* (Dublin: Attic Press, 1992), 107–18, on 112.

35.
John Horgan, ‘Sugaring the pill’, *Fortnight*, 6, (4 December 1970), 9.

36.
‘One woman’s experience’, *Wicca*, 1977, 8 [Attic Press/Róisín Conroy archive, University College Cork: BL/F/AP/1498/3].

37.

Máire Mullarney , What About Me? A Woman for Whom ‘One Damn Cause’ Led to Another (Dublin: Town House, 1992), 161.

38.

Declan Meagher and Dermot MacDonald , ‘A Hospital Family Planning Service’, Journal of the Irish Medical Association, (December 1967), 443–445.6079096

39.

*Ibid*.

40.
‘Undercover on the Pill’, *Woman’s Way*, 1 June 1973, 8–9.

41.
IFPA Annual reports for 1972, 1973, 1974, 1976.

42.
Mary Maher, ‘A Short History of the Pill in Ireland’, *Irish Times*, 14 March 1968, 8.

43.

*Ibid.*, 8.

44.
Maher, *op. cit.* (note 42), 8.

45.
Maher, *op. cit.* (note 42), 8.

46.
For more on the history of *Humanae Vitae*and how the encyclical was received and engaged with in a variety of countries, see: Harris, *op. cit.* (note 15). On reactions from the medical profession in Dublin, see: Foley, *op. cit.* (note 8).

47.
Hug, *op. cit.* (note 6), 86.

48.

*Journal of the Irish Medical Association*, 62, 382 (April 1969), 124–5.

49.
Agata Ignaciuk, ‘Love in the time of El Generalisimo: debates about the pill in Spain before and after *Humanae Vitae*’, in Harris *op. cit.* (note 15), 229–50, on 243.

50.

*Op. cit.* (note 48).

51.
Monica McEnroy, ‘The Contraceptive Pill in Ireland: Personal Involvement’, *Woman’s Way*, 23 September 1966, 9.

52.

*Ibid.*, 9.

53.
McEnroy, *op. cit.* (note 51), 9.

54.
Monica McEnroy, ‘Family Planning’, *Woman’s Way*, week ending Friday 1 March 1968, 19.

55.
Agata Ignaciuk, Teresa Ortiz-Gómez and Esteban Rodríguez-Ocaña, ‘Doctors, women and the circulation of knowledge of oral contraceptives in Spain, 1960s–1970s’ in Teresa Ortiz-Gómez and María Jesús Santesmases (eds), *Gendered Drugs and Medicine: Historical and Socio-cultural Perspectives* (Farnham: Ashgate, 2014), 133–52, on 141.

56.

Eimer Philbin Bowman , ‘Sexual and Contraceptive Attitudes and Behaviour of Single Attenders at a Dublin Family Planning Clinic’, Journal of Biosocial Science, 9, 4 (October 1977), 429–445 , on 435.91489010.1017/s0021932000011275

57.
‘Marriage Guidance’, *Woman’s Way*, 5 July 1968, 27.

58.

*Op. cit.* (note 40).

59.
See: Leanne McCormick, ‘“No Sense of Wrongdoing”: Abortion in Belfast, 1917–1967’, *Journal of Social History*, 49, 1 (2015), 125–48.

60.
Oral history interview with Ruth Riddick, 05-02-2017.

61.

Nuala Fennell , Political Woman: A Memoir (Dublin: Currach Press, 2009), 79.

62.
Oral history interview with Joanne O’Brien, 31-01-2017.

63.
Oral history interview with Betty Purcell, 16-01-2017.

64.
Oral history interview with ‘Maria’, 02-04-2018.

65.
Hilliard, *op. cit.* (note 14), 36.

66.
Marian Larragy cited in Ann Rossiter, *Ireland’s Hidden Diaspora: The ‘Abortion Trail’ and the Making of a London-Irish Underground, 1980–2000* (London: IASC Publishing, 2009), 146.

67.
‘Letters to the editor’, *Irish Times*, 11 January 1966, 14.

68.
Foley, *op. cit.* (note 8), 159.

69.
See: Connolly, *op. cit.* (note 11), 111–29.

70.
‘Family planning’, *Woman’s Way*, 8 March 1968, 20.

71.
Oral history interview with Ruth Riddick, 05-02-2017.

72.
See for example: Diane Gervais and Danielle Gauvreau, ‘Women, Priests, and Physicians: Family Limitation in Quebec, 1940–1970’, *Journal of Interdisciplinary History*, xxxiv: 2 (Autumn, 2003), 293–314; Rusterholz, *op. cit.* (note 5), 41–68; Angelo Somers and Frans van Poppel, ‘Priest, Parishioner and Posterity: A Dutch Urban Legend or Historical Fact?’, *The History of the Family*, 15, 2 (2010), 174–90.

73.
Hilliard, *op. cit.* (note 14), 37.

74.
‘Alteration of Law would be “A curse Upon our Country”: Archbishop’s Pastoral’, *Irish Times*, 29 March 1971, 11.

75.
‘Alteration of law would be “a curse upon our country”’, 11.

76.
‘Contraception: What Do You Think?’, *Irish Times*, 22 December 1970, 6.

77.
Letter from Dublin woman to Archbishop John Charles McQuaid, March 1971. [Dublin Diocesan Archives: McQuaid Papers: Public Affairs: XX/89/3].

78.

*Op. cit.* (note 40).

79.
June Levine, *Sisters: The Personal Story of an Irish Feminist* (Cork: Attic Press, new edition, 2009), 67.

80.
Hilliard, *op. cit.* (note 14), 38.

81.
Hilliard, *op. cit.* (note 14), 38.

82.
Nuala Fennell, *Political Woman: A Memoir* (Dublin: Currach Press, 2009), 79.

83.
Leane, *op. cit.* (note 14), 43.

84.
Gervais and Gauvreau, *op. cit.* (note 72), 313; Leslie W. Tentler, *Catholics and Contraception: An American History* (Ithaca: Cornell University Press, 2008), 244.

85.
Rosita Sweetman, *On Our Backs: Sexual Attitudes in a Changing Ireland* (London: Pan Books, 1979), 157.

86.
Rusterholz, *op. cit.* (note 5), 51.

87.
McEnroy, *op. cit.* (note 51), 9.

88.
Máire Mullarney, *What About Me? A Woman for Whom ‘One Damn Cause’ Led to Another* (Dublin: Town House, 1992), 159.

89.
As Cloatre and Enright have pointed out, scholarly focus on the story of difficult institutional reform with regard to the law on contraception in Ireland has meant that such stories are often marginalised. Cloatre and Enright, *op. cit.* (note 13), 2.

90.
See: Connolly, *op. cit.* (note 11). See also: Yvonne Galligan, *Women and Politics in Contemporary Ireland: From the Margins to the Mainstream* (London: Pinter, 1998); Linda Connolly, ‘The consequences and outcomes of second-wave feminism in Ireland’, in Linda Connolly and Niamh Hourigan (eds), *Social Movements and Ireland* (Manchester: Manchester University Press, 2006), 58–85, Ailbhe Smyth, ‘The Women’s Movement in the Republic of Ireland, 1970–1990’, in Ailbhe Smyth (ed.), *Irish Women’s Studies Reader* (Dublin: Attic Press, 1993), 245–69. For recent studies of the women’s activism around reproductive rights, see Mary Muldowney, ‘Breaking the silence: pro-choice activism in Ireland since 1983’, in Jennifer Redmond, Sonja Tiernan, Sandra McAvoy and Sonja Tiernan (eds), *Sexual Politics in Ireland* (Dublin: Irish Academic Press, 2015), 127–53; Mary McAuliffe, ‘“To change society”: Irishwomen united and political activism, 1975–1979’, in: Mary McAuliffe and Clara Fischer (eds), *Irish Feminisms: Past, Present and Future* (Dublin: Arlen House, 2015), 85–102; Sandra McAvoy, ‘From anti-amendment campaigns to demanding reproductive justice: the changing landscape of abortion rights activism in Ireland 1983–2008’, in J. Scwheppe (ed.), *The Unborn Child, Article 40.3.3 and Abortion in Ireland: Twenty-Five Years of Protection?* (Dublin: Liffey Press, 2008), 15–45; Anne Speed, ‘The struggle for reproductive rights: a brief history in its political context’, in Ailbhe Smyth (ed.), *The Abortion Papers* (Dublin: Attic Press, 1992), 85–98.

91.
Watkins, *op. cit.* (note 22), 119.

92.
Watkins, *op. cit.* (note 22), 104.

93.
Watkins, *op. cit.* (note 22), 3.

94.
Anne Speed, ‘The struggle for reproductive rights: a brief history in its political context’, in Ailbhe Smyth (ed.), *The Abortion Papers* *op. cit.* (note 90), on 91.

95.
Connolly, *op. cit.* (note 11), 112.

96.
For a detailed examination of the IWLM, see: Connolly, *op. cit.* (note 11).

97.
Yvonne Galligan, *Women and Politics in Contemporary Ireland: From the Margins to the Mainstream* (London: Pinter, 1998), 52.

98.
Mary Kenny, ‘Recollections of the Irish Women’s Liberation Movement’, *History Ireland*, 23, 5 (September/October 2015).

99.
Oral history interview with Dr. Eimer Philbin Bowman, 12-01-2017.

100.
See: Connolly, *op. cit.* (note 11), 111–29 and Anne Stopper, *Mondays at Gaj’s: The Story of the Irish Women’s Liberation Movement* (Liffey Press, 2005).

101.
‘Women’s Lib defy law on contraceptives’, *Evening Herald*, 22 May 1971, 1.

102.
‘Abortion, Contraception and Liberation…’, *Fownes Street Journal,*1, 7–8 (December 1972–January 1973), no page number.

103.

*Ibid.*, 4.

104.
‘Feminism by Norah Kelly’, *Fownes Street Journal*, 2, 3 (December 1973), 2–3.

105.
Connolly, *op. cit.* (note 11), 129.

106.
Connolly, *op. cit.* (note 11), 130-1.

107.
Irishwomen United Contraception Workshop, 9–10 May 1975 [Attic Press/Róisín Conroy archive, University College Cork, BL/F/AP/1177/24].

108.
For a detailed discussion of the CAP, see: Laura Kelly, ‘Irishwomen United, the Contraception Action Programme and the Feminist Campaign for Free, Safe and Legal Contraception in Ireland, *c.*1975–81’, *Irish Historical Studies*, 43, 164 (2019), 269–97.

109.
Minutes of the Contraception Action Programme meeting, 22 June 1976 [Attic Press/Róisín Conroy archive, University College Cork: BL/F/AP/1177/21].

110.
Statement by Irishwomen United [Attic Press/Róisín Conroy archive, University College Cork: BL/F/AP/1177/23].

111.
‘Contraception: The Slot Machine Government’, *Banshee, Journal of Irishwomen United*, 1, 1 (1976), 5.

112.
Roisin Boyd, ‘Contraception…who to believe’, *Wicca*, 1977, 10 [Attic Press/Róisín Conroy archive, University College Cork: BL/F/AP/1498/3].

113.
Oral history interview with Anne Speed, 16-11-2017.

114.
Oral history interview with Ruth Torode, 11-01-2017.

115.
‘One woman’s experience’, *Banshee*, 6 (1977), 11 [Attic Press/Róisín Conroy archive, University College Cork: BL/F/AP/1498/3].

116.

*Ibid.*, 8.

117.
‘Natural Methods of Birth Control?’, *Banshee*, 6 (1977), 11.

118.

*Ibid*.

119.
‘Contraceptive Bill “The Tip of the Iceberg”’, *Irish Times*, 15 November 1973, 9.

120.
Irish Family League, *Is Contraception the Answer?* (Dublin: Irish Family League, 1974), 22.

121.
‘Fighting the “Lobby”’, *Irish Press*, 8 October 1980 [Attic Press/Róisín Conroy archive, University College Cork: BL/F/AP/846/16].

122.
Irish Family League, *op. cit.* (note 120), 19.

123.
Irish Family League, *op. cit.* (note 120), 5.

124.
Irish Family League, *op. cit.* (note 120), 6.

125.
Irish Family League, *Why You Should Oppose Contraception* (Dublin: Irish Family League, 1976).

126.
Irish Family League, *op. cit.* (note 120), 7.

127.
‘Letters to the Editor: The Contraceptives Bill’, *Irish Times*, 7 August 1974, 11.

128.
‘Letters to the Editor: No Sex Please’, *Irish Times*, 24 December 1976, 9.

129.
‘Letters to the Editor: Family Planning’, *Irish Times*, 4 January 1977, 9.

130.
‘Letters to the Editor: Family Planning’, *Irish Times*, 11 January 1977, 9.

131.
Irish Family League, *Alert: Oral Contraceptive* (Dublin: Irish Family League), 2.

132.
‘Letters to the Editor: Family Planning’, *Irish Times*, 3 November 1975, 9.

133.
‘Letters to the Editor: The Pharmacist’s Duty’, *Irish Times*, 24 November 1976, 11.

134.

*Ibid*.

135.

*Op. cit.* (note 133).

136.
‘Letters to the Editor: Availability of Contraceptives’, *Irish Times*, 13 February 1976, 6.

137.

*Ibid*.

138.
‘Letters to the Editor’, *Irish Times*, 29 December 1976, 9.

139.
‘Letters to the Editor’, *Irish Times*, 13 June 1978, 13.

140.
‘Students’ Call’, *Irish Press*, 29 December 1975, 6.

141.
Róisín Conroy, ‘A Feminist’s Opinion’, *Irish Family Planning Association newsletter*, (January 1979), 3 [Attic Press/Róisín Conroy archive, University College Cork: BL/F/AP/1291/20].

142.
‘Report of the Committee set up by the Executive of the Irish Medical Association to Advise on the Hazards and Side-effects of Ovulation Suppressants’, *Journal of the Irish Medical Association* (supplement), 71, 2 (February 1978).640807

